# Biological Risk Factors for Suicidal Behavior in Children and Adolescents: A Narrative Review

**DOI:** 10.3390/children13030356

**Published:** 2026-02-28

**Authors:** Martin Vatrál, Juraj Jurík, Barbora Katrenčíková, Jana Muchová, Zdeňka Ďuračková, Jana Trebatická

**Affiliations:** 1 Department of Paediatric Psychiatry, The National Institute of Children’s Diseases, Faculty of Medicine, Comenius University, Limbová 1, 833 40 Bratislava, Slovakia; vatral3@uniba.sk (M.V.); juraj.jurik@nudch.eu (J.J.); jana.trebaticka@fmed.uniba.sk (J.T.); 2 Faculty of Medicine, Institute of Medical Chemistry, Biochemistry and Clinical Biochemistry, Comenius University, Sasinkova 2, 813 72 Bratislava, Slovakia; katrencikov2@uniba.sk (B.K.); jana.muchova@fmed.uniba.sk (J.M.)

**Keywords:** youth suicidality, suicidal behavior, neurobiology, inflammation, biomarkers, kynurenine pathway, neuroplasticity, ketamine

## Abstract

**Highlights:**

**What are the main findings?**

- Biological risk factors contribute to suicidality in interaction with established psychosocial risk factors.
- Key neurobiological risk factors of suicidality include dysfunction in the serotonin system, impaired neuroplasticity (marked by glutamate–GABA imbalance and reduced BDNF), and dysregulation of stress-response pathways, including the HPA axis and chronic inflammation.

**What are the implications of the main findings?**

- A deeper understanding of these biological mechanisms is essential for the development of targeted prevention and intervention strategies tailored to the unique developmental needs of children and adolescents.
- Future research must prioritize longitudinal data and integrative, developmentally informed models to clarify causal mechanisms and overcome current challenges in translating biological findings into clinical practice.

**Abstract:**

Suicidal behavior in children and adolescents is a major global public health issue, and suicide is one of the leading causes of death in this age group. While psychosocial determinants of suicidality are well established, understanding its biological risk factors is crucial for targeted prevention and treatment. This review presents a narrative synthesis of recent literature examining current evidence on the biological mechanisms that contribute to youth suicidality. Genetic liability plays a substantial role, often interacting with environmental stressors. Key neurobiological factors include dysfunction of the serotonin system and impaired neuroplasticity, characterized by a glutamate–gamma-aminobutyric acid imbalance and reduced brain-derived neurotrophic factor. Psychosocial stress appears linked to these changes through several pathways, including dysregulation of the hypothalamic–pituitary–adrenal axis, chronic low-grade inflammation, oxidative stress, and activation of the kynurenine pathway. Neurodevelopmental conditions like autism spectrum disorders and attention deficit hyperactivity disorder, as well as sleep disturbances, may further increase risk. While therapeutic agents such as ketamine and lithium target these neurobiological systems, evidence for their anti-suicidal efficacy in youth remains limited, with only a small number of randomized controlled trials conducted in pediatric populations. Biological research offers valuable insights, but the use of varied study methods and a lack of longitudinal data complicate its translation into clinical practice. Future studies should employ integrative, developmentally informed models to elucidate causal mechanisms and inform more effective interventions.

## 1. Introduction

Suicidal behavior among children and adolescents is a critical public health concern with severe societal consequences. Suicides consistently rank among the leading causes of death for adolescents in Europe and the United States. Although overall rates have declined in some regions over the past decades, trends vary substantially across countries, and recent data indicate stabilization or increases in certain populations [1,2,3,4]. A common misconception persists that children are incapable of contemplating suicide due to their developmental stage. However, research demonstrates that even school-age children under twelve years of age can experience suicidal thoughts and behaviors [3,5]. These findings challenge age-based assumptions about suicidality and underscore the need for developmentally informed research and heightened clinical vigilance in child and adolescent mental health care.

Suicidal behavior is not attributable to a single cause but rather arises through the dynamic interaction of multiple risk determinants, encompassing biological, psychological, social, and environmental conditions [6]. While the influence of psychosocial stressors is widely acknowledged, these factors likely exert their effects in part through underlying neurobiological processes that shape stress responsivity, emotional regulation, and behavioral control. A more profound comprehension of the underlying biological mechanisms is therefore crucial for the development of truly effective prevention and intervention strategies [7,8,9].

This narrative review synthesizes current evidence on biological risk factors for suicidality in children and adolescents, with a focus on neurobiological mechanisms, genetic and epigenetic influences, and peripheral biomarkers. The findings aim to highlight knowledge gaps and potential opportunities for prevention and intervention.

### Literature Identification and Scope

Literature was identified primarily through targeted searches of PubMed using combinations of keywords related to suicidality, children and adolescents, risk factors, and biological or neurobiological mechanisms. Searches were complemented by manual screening of reference lists from relevant reviews and seminal papers. We focused predominantly on literature published within the past decade, with particular emphasis on studies from the last five years, while incorporating earlier foundational work where necessary to contextualize core mechanistic frameworks. Priority was given to studies with pediatric samples and to higher-level evidence, including systematic reviews, meta-analyses, umbrella reviews, and longitudinal or population-based designs where available. Given the limited availability of pediatric neurobiological data in several domains, findings from translational and adult suicide research were frequently incorporated to inform biological frameworks. This integration was undertaken with acknowledgment of the developmental limitations inherent in extrapolating adult findings to younger populations.

## 2. Suicidality in Slovakia, Age, and the Gender Paradox

Across Europe, suicide mortality has declined over the past decade; however, substantial regional disparities persist. Some Eastern European countries (including Slovakia) continue to report higher age-adjusted rates than Western Europe [10]. According to WHO data, Slovakia falls into the high suicide mortality category, especially among older men [11].

The National Health Information Centre (NCZI) has monitored suicidality in Slovakia since 2008. Male suicidality follows a relatively stable age-dependent pattern, with low mortality in boys and adolescents but a steep increase across adulthood; although suicide attempts are less frequent among males generally, mortality rates consistently exceed those observed in females. In contrast, female patterns are more dynamic. Girls aged 0–14 years exhibited consistently low rates of suicide and attempts until a marked increase in the rate of attempts during the late 2010s and the COVID-19 pandemic period. Adolescent females aged 15–19 have persistently shown the highest suicide attempt rates of any group, with pronounced rises during these years. Completed suicides in this group remain comparatively uncommon despite year-to-year variability, including a transient peak in 2021 [12].

International evidence suggests that some of these patterns may reflect broader global trends associated with the COVID-19 pandemic. In Catalonia, Spain, a population-based registry recorded a 25% overall increase in adolescent suicide attempts during the first pandemic year. The rise was driven almost entirely by girls, while boys’ rates remained stable [13]. Similar patterns emerged in the United States, where emergency visits for suspected suicide attempts among adolescent girls were 51% higher in early 2021 compared with the same period in 2019, in contrast to only minimal change among boys [14]. Global analyses likewise point to sustained increases in female youth suicide mortality in several countries (including the United Kingdom, South Korea, and Japan) during the years preceding and overlapping with the pandemic [2].

The data [12] also underscore a gendered dimension to suicide risk, where adolescent girls are more prone to attempts, while older men are more likely to die by suicide. This reflects the well-established “gender paradox” in suicidality, defined as the higher prevalence of non-fatal suicide attempts among females alongside higher suicide mortality among males across age groups [15,16,17,18].

The lethality of a suicide attempt is shaped by several factors, including access to the chosen method, awareness of its fatal potential, familiarity with its use, and the influence of substances such as alcohol or drugs [15,17]. Studies of psychiatric inpatients further indicate that those who employ highly lethal means often share identifiable demographic and clinical characteristics, though not all investigations have observed sex differences in lethality [15]. Research more broadly suggests that men are more likely to employ highly lethal methods, such as firearms or jumping in front of vehicles, whereas women more often attempt self-poisoning [15,17,18].

Taken together, these findings show that suicidal behavior varies systematically across gender and age. For research on children and adolescents, the key implication is to investigate why suicidal behavior is more prevalent in youth, even if it less often results in death compared with older populations. These distinct age-related patterns raise the question of whether specific neurobiological vulnerabilities, such as an immature stress response, might play a role in driving the elevated risks observed in young people. However, this represents a proposed explanatory framework rather than established causal evidence.

## 3. Psychological, Social, and Environmental Risk Factors for Suicidal Behavior

**Figure 1:**
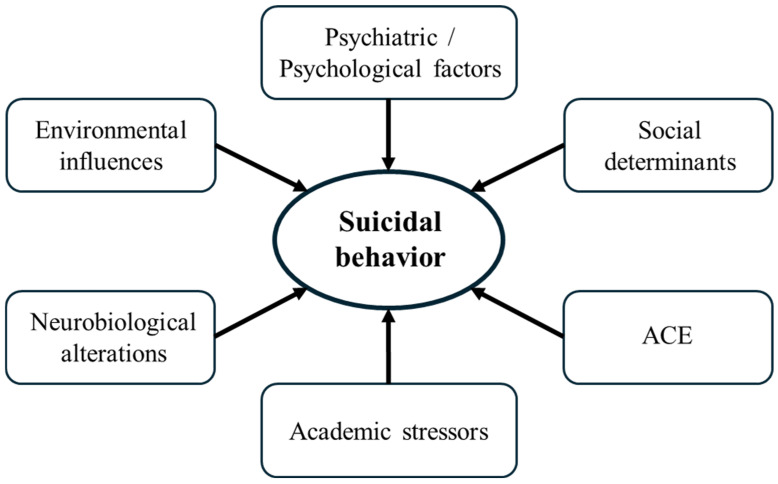
Risk factors for suicidal behavior. ACE = adverse childhood experiences.

Suicidal behavior in children and adolescents is shaped by biological, psychological, social, and environmental influences. Due to the developmental immaturity of young people, which includes ongoing identity formation, reliance on caregivers, and limited emotion regulation, they are particularly vulnerable to these stressors [19]. Based on this, contemporary research adopts a socio-ecological perspective, showing how risks across individual, relational, community, and societal domains accumulate and interact to trigger suicidal thoughts and behaviors in youth [20]. Some of the main risk factors for suicidal behavior are shown in Figure 1.

Psychiatric and psychological factors remain among the most robust predictors of suicidality. Depressive and anxiety disorders, as well as self-harming behavior, are consistently implicated in suicidal processes among adolescents [19,21,22]. Emotion regulation difficulties, manifesting as low self-esteem, hopelessness, and poor coping strategies, have also emerged as strong correlates of suicidal behavior in youth populations [5,20,23,24,25]. Notably, sleep disturbances constitute a potentially modifiable risk factor. In a dose–response meta-analysis of five studies in adolescents, each additional hour of sleep was associated with an approximately 11% lower odds of suicide plans, although the included studies were predominantly cross-sectional [20,26].

Research consistently highlights the profound impact of adverse childhood experiences on suicidality. Childhood trauma, abuse, family dysfunction, and related familial stressors significantly elevate suicidal risk among adolescents. Indeed, parental neglect, conflict, low socioeconomic status, and unstable family environment are frequently cited as foundational contributors to suicidal vulnerability [20,23,24,25].

Academic stressors have been increasingly recognized as significant contributors to suicidality among children and adolescents, operating through complex psychosocial and neurobiological pathways. Qualitative and systematic reviews show that pressures such as excessive workload, high-stakes examinations, and competitive educational environments are linked with greater suicidal ideation and attempts in youth populations [27,28]. These stressors rarely occur in isolation, often compounding existing vulnerabilities such as depression, anxiety, or bullying, thereby amplifying the risk of self-harm behaviors [29,30].

Evidence from Asia further highlights the magnitude of this problem. In Japan, stress related to school records and academic courses has been identified as a major predictor of suicidality among adolescents [31]. In South Korea, academic achievement stress is strongly linked with suicidal ideation in young people [32], and in South Asia, competitive entrance examinations have been documented as drivers of suicidal impulses among Bangladeshi students [33]. While cultural dynamics differ, similar challenges are present in Slovakia and Central Europe, where academic demands and exam-related pressures have been shown to contribute to negative effects like elevated levels of stress, anxiety, and depression among students [34,35]. However, across these studies, depressive symptoms are typically examined as co-occurring factors rather than consistently controlled for in a manner that isolates the independent contribution of academic stress, and therefore, the extent to which academic stress confers risk beyond depression remains to be clarified.

Furthermore, broader social determinants such as racial and ethnic minority status, socioeconomic marginalization, and associated stigma exacerbate risk. Multi-racial or marginalized youth groups experience higher rates of suicide-related behaviors [36]. For example, studies focusing on Black children show how racism, poverty, and limited access to care interact to increase suicide risk. This highlights the dangerous impact of facing multiple, overlapping disadvantages [37]. In addition, migration-related stressors likewise heighten vulnerability, as discrimination and exclusion erode mental health and increase suicidality [19,20].

Evidence shows that sexual and gender minority adolescents are at particularly high risk as well, driven by stigma, discrimination, and hostile environments, with risks most significant in societies where laws and norms are restrictive [20,38]. Religion further conditions outcomes. While faith communities may foster resilience, rigid or exclusionary doctrines can intensify marginalization, especially for lesbian, gay, bisexual, transgender, and questioning (LGBTQ) youth [39].

At the same time, broader societal determinants such as economic inequality, unemployment, and political environments represent structural risk factors that shape population-level suicide patterns [40]. In other words, the risk is further influenced by demographic and contextual variables [41]. For instance, during the COVID-19 pandemic, urban residence and family job loss were linked with greater adolescent suicide attempts [27].

From a practical standpoint, access to highly lethal means, such as firearms or toxic substances, is a major determining factor of whether suicidal crises in adolescents prove fatal [1,20]. Reviews consistently show that the availability of these methods, combined with unsafe storage, increases risk of suicide, while secure storage practices and policy restrictions are associated with reduced mortality [1,19]. Limiting access to lethal means is therefore considered a well-supported and effective strategy for preventing youth suicide [1,20].

Current research indicates that psychosocial stressors such as bullying may influence suicidality through neurobiological alterations, including hyperactivation of the hypothalamic–pituitary–adrenal (HPA) axis, serotonergic dysregulation, and reduced brain-derived neurotrophic factor (BDNF) levels. This proposed mechanistic framework is supported by converging evidence from human neuroimaging studies showing stress-related structural and functional brain changes, peripheral biomarker research examining HPA axis reactivity and neuroinflammatory markers, and complementary findings from animal models of chronic stress [42,43]. Given the accumulating body of scientific evidence in this area, this review focuses on biological risk factors for suicidal behavior in children and adolescents.

## 4. Biological Risk Factors for Suicidal Behavior

### 4.1. Genetic and Familial Factors

Genetic factors play a substantial role in shaping the risk of suicidal behavior in children and adolescents [44,45]. Studies comparing relatives and twins indicate that genetic liability plays a significant role in suicidality, with heritability estimates for suicidal thoughts, plans, and attempts ranging between 30 and 55 percent [46]. Moreover, the risk to offspring of suicidal parents remains elevated even when controlling for shared environmental factors, suggesting that both inherited genetic liability and other familial influences, such as exposure to suicidal behavior of caretaking adults, independently contribute to youth vulnerability [23,47,48,49,50,51].

Genetic research on suicidality has historically focused on genes regulating stress responses and mood pathways, with SLC6A4 (the serotonin transporter) and BDNF among the most studied [52,53]. Early findings suggested that the short allele of 5-HTTLPR and the BDNF Val66Met variant might increase vulnerability to suicidal behavior, particularly under stress; however, subsequent large-scale investigations and analyses of genome-wide association studies (GWAS) have demonstrated that these effects are modest, highly context-dependent, and not consistently replicated [52,54,55]. Genes influencing the HPA axis, such as FKBP5, have also gained attention for their role in gene–environment interactions, especially when childhood trauma is present [52,53]. Foundational GWAS later confirmed that suicide risk is not attributable to individual common variants of large effect. This has decisively shifted the field toward polygenic risk models, which acknowledge that suicide’s heritability likely arises from the cumulative impact of many small genetic variations interacting with contextual stressors [52,56]. However, although polygenic approaches have advanced etiological understanding, their current predictive utility in youth populations remains limited [52,54,56].

Recent findings using polygenic risk scores also suggest that genetic liability can intensify the effects of adverse experiences. In a population-based cohort of 2571 UK adolescents from the Avon Longitudinal Study of Parents and Children (ALSPAC), suicidal ideation at age 17 was associated with polygenic risk for suicide attempts in girls but not boys. Notably, a gene–environment interaction was observed in girls, whereby drug use was more strongly associated with suicidal ideation among those with elevated genetic liability. This interaction between genes and environment indicates that inherited predispositions may amplify the impact of negative psychosocial exposures early in life [44].

### 4.2. Neurotransmitter Dysregulation and Impaired Neuroplasticity

#### 4.2.1. Serotonergic System Dysfunction

Dysfunction within the central serotonin (5-HT) system is one of the most consistently identified neurobiological correlates of suicidal behavior [53,57]. Postmortem studies of suicide victims reveal a decreased density of serotonin transporters in key brain regions such as the ventrolateral prefrontal cortex, which may impair the brain’s capacity for top-down emotional and decision-making control [43,53]. However, most postmortem evidence derives from adult samples, as direct pediatric postmortem studies are rare due to ethical and methodological limitations [58,59]. The deficit in serotonin signaling is further supported by findings of low concentrations of the serotonin metabolite 5-hydroxyindoleacetic acid (5-HIAA) in the cerebrospinal fluid (CSF) of individuals who attempt suicide [53,60]. One of the possible ways the brain appears to compensate for this low serotonergic activity is through the upregulation of postsynaptic 5-HT2A receptors and presynaptic 5-HT1A autoreceptors in the prefrontal cortex and raphe nucleus, respectively [43,61].

Fundamental disruptions in serotonin signaling may be especially consequential because of heightened neural plasticity in adolescents, creating a significant vulnerability for the development of suicidal behaviors in response to stress [53,60,61].

The clinical relevance of serotonergic dysfunction in suicidality is underscored by evidence from antidepressant treatment studies. Selective serotonin reuptake inhibitors (SSRIs), medications that block serotonin reuptake and thereby increase its synaptic presence, are widely used as an effective first-line therapy for depression. As mentioned earlier, depressive disorders are associated with a heightened risk of suicidality [62,63,64]. However, large-scale cohort and meta-analytic data indicate that initiation of SSRIs in adolescents and young adults may be associated with a transient increase in suicidal behavior, particularly in the early weeks of treatment. In pooled analyses of pediatric trials, the relative risk of suicidal ideation or behavior with SSRIs has been estimated at approximately 1.6 compared with placebo, with absolute event rates generally below 10% and no demonstrated increase in completed suicide [62,65]. This observation should be interpreted within a broader risk–benefit framework, as effective treatment of depression remains central to suicide prevention [62].

Serotonin’s role in suicide risk is therefore complex. SSRIs act on affective symptoms such as depressed mood and anxiety, with subsequent improvements in cognitive domains like guilt or loss of interest, which are closely linked to hopelessness and suicidal thinking [66]. Treatment-emergent suicidal ideation and its worsening are not uniform phenomena. They are influenced by age, baseline severity, genetics, and comorbid conditions [65]. These findings highlight both the importance of serotonin-targeting drugs in therapeutic strategies and the clinical necessity of close monitoring in vulnerable populations.

#### 4.2.2. Dopaminergic Pathways in Reward and Cognition

The role of the dopaminergic system in suicide is less clearly defined than that of serotonin. However, its involvement in reward processing, motivation, and executive function suggests it may contribute to psychological traits underlying suicide risk [53,67]. A diminished capacity to process rewards may contribute to reduced sensitivity to positive reinforcement as well as hopelessness, one of the most consistent psychological predictors of suicidal behavior [53,60,61].

Some studies report reduced dopaminergic activity in suicide attempters, for example, a blunted growth hormone response to the dopamine agonist apomorphine. However, postmortem studies have generally not found consistent alterations in dopamine receptor densities or dopamine transporter availability in suicide victims [53]. Findings on dopamine metabolites have not been uniform. Reductions in 3,4-dihydroxyphenylacetic acid (DOPAC) and elevations in homovanillic acid (HVA) were reported in specific brain regions, but not consistently so across samples [53,68]. A more recent meta-analysis found significantly reduced CSF levels of HVA in suicide attempters, indicating decreased dopamine turnover. The same analysis noted that the dopaminergic system has received relatively limited attention as a contributor to suicide vulnerability. Its findings were based on a small number of studies with predominantly adult samples [69]. As a result, direct inference for pediatric populations remains limited.

Taken together, these results suggest that if dopaminergic dysfunction contributes to suicide risk, its effects are likely subtle and context-dependent. One hypothesis proposes that it may act in coordination with other neurotransmitter abnormalities and dysfunction in frontal–subcortical connections or cortico–striato–thalamic circuits, potentially impairing decision-making and cognitive control in vulnerable individuals [53,60,70].

#### 4.2.3. Glutamatergic/GABAergic Imbalance and Neuroplasticity

Converging evidence indicates that suicidal behavior is associated with a fundamental imbalance between the brain’s primary excitatory (glutamate) and inhibitory (gamma-aminobutyric acid, GABA) systems. This evidence derives predominantly from adult postmortem and translational studies, with limited direct evidence in pediatric populations [43,60,61]. Studies of postmortem brain tissue from individuals who died because of suicide have found altered expression of genes associated with glutamatergic and GABAergic neurotransmission in the prefrontal cortex and hippocampus [53,61]. This dysregulation between excitatory and inhibitory processes is considered a critical component of a broader deficit in neuroplasticity (the brain’s ability to adapt and reorganize itself) [43,61].

BDNF is a key molecule for neuronal survival, growth, and synaptic plasticity [71,72]. Reduction in the levels of BDNF is frequently found in the brains and peripheral blood of individuals who exhibit suicidal behaviors [43,61]. Whether these changes reflect a stable trait vulnerability or a state-dependent alteration associated with acute stress or suicidal crises remains unclear [43,60,72]. The deficit in BDNF is believed to be a downstream consequence of multiple factors, including hyperactivity of the HPA stress axis and inflammation-induced glutamatergic overstimulation. These can be neurotoxic. Impairments in neuroplasticity may, in this way, underlie the cognitive deficits in learning and memory often observed in individuals who attempt suicide [43,53,60,61]. The rapid antisuicidal effects of ketamine, an N-methyl-D-aspartate (NMDA) glutamate receptor antagonist, further reinforce this link. Its mechanism of action is thought to involve modulation of glutamatergic transmission and engagement of multiple plasticity-related pathways, including AMPA receptor-dependent signaling, BDNF-TrkB activation, and mammalian target of rapamycin (mTOR)-related protein synthesis. Collectively, these processes are thought to reduce excitotoxic stress and promote restoration of synaptic plasticity, partly through the release of BDNF [73,74].

**Figure 2:**
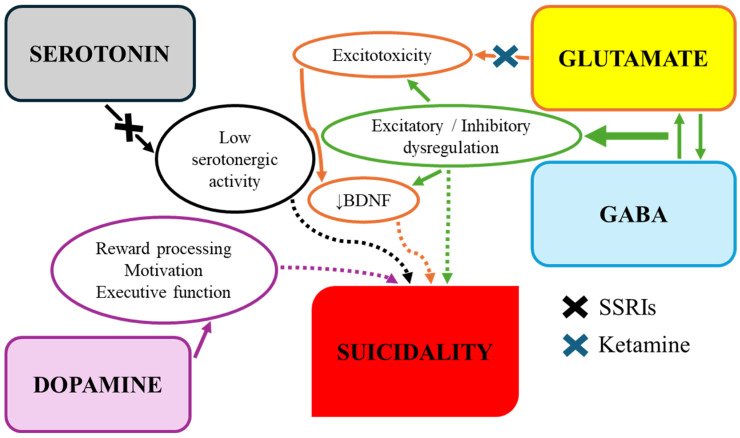
Possible mechanisms of neurotransmitter action in suicidality and potential for therapeutic interventions (indicated by X symbols). Solid arrows represent established neurobiological relationships. Dotted arrows denote hypothesized or primarily associative links to suicidality. SSRIs = selective serotonin reuptake inhibitors; GABA = gamma-aminobutyric acid; ↓BDNF = reduced brain-derived neurotrophic factor levels.

Figure 2 illustrates the interrelationships of neurotransmitters implicated in the pathophysiology of suicidal behavior as well as potential pharmacological interventions.

### 4.3. Stress, Inflammation, and Oxidative Imbalance

Stress-related biology is among the most extensively studied mechanisms linking adversity to suicidal behavior in youth. Beyond the previously discussed neurotransmitters, the pathophysiology of suicidality may also involve disturbances in the HPA axis and the kynurenine pathway, immune dysregulation, neuroinflammation, and redox imbalance [9,43,60,75,76].

#### 4.3.1. HPA Axis Dysregulation

A widely accepted biological framework for suicide emphasizes the interplay between stress-response systems and inflammatory mechanisms, with the HPA axis as a central component [43,60,61]. Postmortem studies indicate chronic activation of the HPA axis in suicide victims, including adrenal hypertrophy, thickened cortical layers, and elevated corticotropin-releasing hormone (CRH) in corticolimbic regions [9,61,77].

Clinical studies add further support, though findings in young populations remain inconsistent. Some report elevated cortisol associated with suicidal behaviors, while others identify blunted cortisol responses to laboratory stress paradigms, suggesting heterogeneity in HPA dysregulation across adolescents [43,53,78]. These apparently contradictory findings may reflect two partially distinct patterns of abnormal HPA axis activity: acute stress-related hyperactivity and chronic stress-associated attenuation.

Consistent with the hyperactivity pattern, in a large Mexican cohort, cortisol concentrations were elevated in people with a history of suicide attempts in comparison to healthy controls. The increase was most pronounced in those with multiple attempts and in attempters who also met criteria for depression [79]. A study of psychiatric inpatients using hair cortisol analysis showed that lower cumulative cortisol output was present before suicide attempts. These patients also exhibited reduced glucocorticoid receptor gene expression together with signs of heightened inflammation, including increased C-reactive protein (CRP) and tumor necrosis factor-α (TNF-α) levels [80]. Beyond cortisol, elevated corticotropin-releasing hormone has been detected in stress-regulatory brain regions [9,81]. In adolescents, an abnormal HPA axis functioning is thought to underlie poor emotion regulation, itself a risk factor for suicidal behavior [82].

Taken together, this body of evidence points to divergent patterns: hyperactivity in some cases and hypocortisolism in others. Such variation suggests that developmental stage, psychiatric comorbidity, and accumulated stress may influence the trajectory of HPA axis changes [9,79,80,83]. Importantly, youth-focused studies demonstrate that lower hair cortisol can differentiate suicide attempters from those with ideation alone, underscoring its potential as a marker of short-term risk [80]. Overall, both high and low HPA activity may reflect dysregulated stress responsivity, in which either exaggerated or blunted cortisol signaling could interfere with adaptive emotion regulation and increase vulnerability to suicidal behavior [9,77,79,80].

#### 4.3.2. Inflammation and Neuroinflammation

Stress-induced alterations in the HPA axis are closely coupled with immune activation. High levels of pro-inflammatory cytokines, notably interleukin-6 (IL-6) and TNF-α, have been detected in the samples of blood, CSF, and post-mortem brain tissue of suicidal individuals [9,61,75,78].

Meta-analytic evidence in major depressive disorder (MDD), largely derived from adult samples, indicates small but consistent peripheral increases, with weighted mean differences of approximately 1–2 pg/mL for IL-6 and 3–4 pg/mL for TNF-α compared with controls [84,85]. Although pediatric-specific data remain more limited and heterogeneous, similar directional elevations have been reported in child and adolescent samples [86]. These cytokines are not merely peripheral markers. They interact with stress-regulatory systems and neurotransmitter pathways, thus shaping core depressive symptoms and potentially influencing treatment [87].

The role of nitric oxide (NO) in suicidal behavior is contentious, as studies yield inconsistent findings [76]. While NO is an important neurotransmitter implicated in depression pathophysiology, some research has detected elevated NO concentrations in depressed patients with suicidal thoughts [88]. Conversely, a recent study in adolescents with MDD found significantly reduced serum NO levels in those with a history of suicide attempts compared with non-attempters. This discrepancy may reflect NO’s dual function, acting as a signaling molecule at low concentrations but as a damaging free radical in excess. Overall, NO alterations may represent context-dependent changes in oxidative stress and neuroinflammatory signaling, with the direction of change varying according to clinical and developmental factors. For these reasons, its clinical significance remains uncertain [76,88].

Beyond peripheral changes, converging neuropathological studies highlight neuroinflammatory processes within the brain. Postmortem analyses of individuals who died by suicide reveal an increased ratio of primed to resting microglia and greater macrophage recruitment in the anterior cingulate white matter, consistent with low-grade neuroinflammation [89]. Microglial activation is of particular interest because these cells regulate local immune responses and, when chronically stimulated, may release neurotoxic mediators that are associated with alterations in white-matter integrity [87,89]. At the molecular level, inflammatory mediators converge on transcriptional regulators, including nuclear factor-κB (NF-κB), which can be activated by cytokine signaling and oxidative stress, potentially sustaining immune responses linked to neuroprogression. Nevertheless, the extent to which these processes contribute causally to suicidal behavior has yet to be clarified [8,88].

Importantly, pediatric studies suggest that these mechanisms are evident from early stages of psychiatric disorders. Children and adolescents with depressive disorders exhibit elevated inflammatory and oxidative stress markers alongside reduced antioxidant defenses, underscoring that immune dysregulation is not limited to adult depression [90,91]. Findings from the Slovak DEPOXIN project provide further support, demonstrating that cytokine activation, redox imbalance, shifts in lipid mediators, and neuroendocrine stress responses collectively define a characteristic biological profile in youth depression [91].

Taken together, current evidence supports a framework in which depression and suicidal behavior involve both systemic and central immune dysregulation. These processes interact with other biological systems to shape vulnerability [84,87,88,89].

#### 4.3.3. Redox Imbalance and Oxidative Stress

Oxidative stress denotes a state in which reactive oxygen and nitrogen species (ROS/RNS) outpace the organism’s antioxidant defense systems. It is increasingly recognized for its role in depression and suicidality. The brain’s high lipid content, oxygen demand, and relatively weak antioxidant buffering make it especially vulnerable to oxidative injury [76,88,92].

Findings from pediatric cohorts with depressive disorders highlight consistent reductions in glutathione peroxidase (GPx) activity [90,91], while results for superoxide dismutase (SOD) are more heterogeneous. Some studies observed no baseline difference from controls [90], others reported decreased SOD activity in depressed youth [93], and recent work found increased SOD among adolescents with suicide attempts, suggesting a possibly compensatory or stress-related response [76]. Such variability may reflect both biological heterogeneity across illness stages and clinical presentations, as well as methodological differences between studies, including assay techniques, biological matrices (e.g., serum vs. plasma or erythrocytes), and the timing of sample collection [76,91,93]. Together, these findings support a role for impaired antioxidant defenses in pediatric depression and suicidality, while indicating that specific enzyme patterns may vary across clinical and methodological contexts.

Markers of oxidative damage, including nitrotyrosine, lipid peroxidation products (for example, 8-isoprostane), and advanced oxidation protein products (AOPP), are elevated in depressed youth and correlate with symptom severity [76,88,92]. Beyond its role in inflammation, the dysregulation of NO metabolism has also been linked to suicidal ideation, further implicating redox imbalance in suicide risk [76,88].

Depressive disorder in children and adolescents is influenced by the ratio of omega-6 (pro-inflammatory properties) to omega-3 (anti-inflammatory properties) fatty acids (FAs) [91]. Intervention trials within the DEPOXIN project show that omega-3 FAs reduce oxidative stress markers, enhance antioxidant defenses, and alleviate depressive symptoms [90]. Recently, higher levels of omega-3 FAs have been associated with reduced risk of self-harm and suicidal ideation. However, these findings derive primarily from observational data with modest effect sizes and low event rates, limiting conclusions about a specific anti-suicidal effect [94]. Additional evidence suggests a positive correlation between cortisol and lipoperoxides, as well as between aldosterone and 8-isoprostane. This ties endocrine stress responses to oxidative injury and may help explain pathways of vulnerability to suicidality [90,91,95].

Redox and inflammatory signaling appear bidirectionally coupled, and redox changes may coincide with neuroimmune alterations observed in individuals who died by suicide and had a depressive disorder [87,88,89].

#### 4.3.4. Kynurenine Pathway

A key downstream consequence of inflammation and oxidative stress is the activation of the tryptophan–kynurenine pathway. Pro-inflammatory cytokines stimulate indoleamine 2,3-dioxygenase (IDO), which diverts tryptophan metabolism away from serotonin synthesis and toward kynurenine production [75,88,96,97]. This process reduces the availability of serotonin precursors while generating neuroactive metabolites that influence glutamatergic neurotransmission [96,97].

Kynurenine can be metabolized into either the neuroprotective kynurenic acid or into neurotoxic derivatives such as 3-hydroxykynurenine and quinolinic acid. Evidence indicates that in depressive states, particularly those associated with suicidal behavior, metabolism shifts toward the neurotoxic branch [75,96,97,98]. In adult cohorts, elevated CSF quinolinic acid (QUIN) has been observed in suicide attempters, accompanied by reduced levels of protective metabolites such as picolinic acid (PIC), resulting in an unfavorable PIC/QUIN ratio [8,75,96,98]. Comparable CSF data in pediatric populations are currently lacking [98,99]. Peripheral blood kynurenine/tryptophan ratio findings, by contrast, have been inconsistently reported in adult samples and may reflect broader inflammatory activation rather than a suicide-specific metabolic shift [96,100].

Ilavská et al. [99] found a disrupted serotonin–kynurenine balance in depressed children and adolescents. At the same time, the DEPOXIN project demonstrated that changes in the kynurenine pathway occur alongside redox and inflammatory alterations [91]. Reviews further emphasize that oxidative stress can amplify IDO activity, thereby reinforcing a feedback loop in which redox and immune signals may drive the pathway [88,91,98].

Overall, the kynurenine pathway provides a possible mechanistic bridge linking peripheral immune activation and oxidative stress to central neurotransmitter imbalances, thereby potentially contributing to both depression and suicide risk.

**Figure 3:**
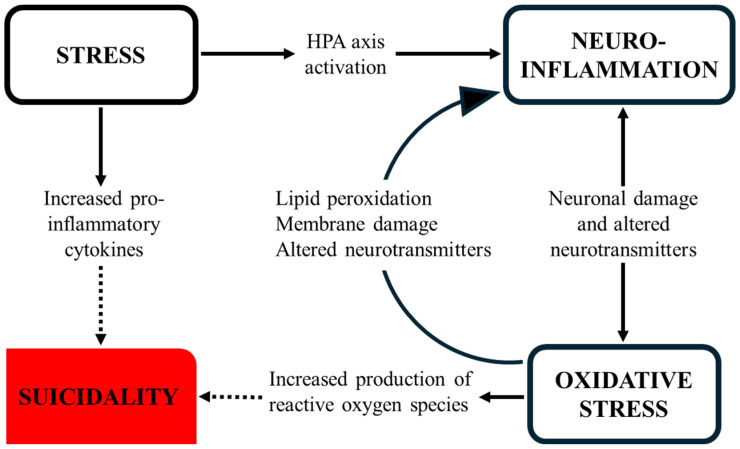
Proposed interrelationships between psychological stress, neuroinflammation, oxidative stress, and suicidality. Solid arrows represent well-described neurobiological relationships. Dotted arrows denote hypothesized or primarily associative links to suicidality. HPA = hypothalamic–pituitary–adrenal.

Figure 3 shows relationships between psychological stress, neuroinflammation, oxidative stress, and suicidality.

### 4.4. Neurodevelopmental and Neurocircuitry Vulnerabilities

Neurodevelopmental disorders are strongly associated with increased risk of suicidal thoughts and behaviors. In a large South London cohort, boys with autism spectrum disorders (ASD) exhibited nearly threefold higher risk of emergency self-harm compared to peers. At the same time, attention deficit hyperactivity disorder (ADHD) was likewise associated with self-harm in both sexes after adjusting for confounders [101]. A recent meta-analysis reported elevated odds of suicidal ideation and attempts in youth with ADHD, although effect sizes varied across study designs, underscoring heterogeneity [102]. Trait-level data further suggest cumulative risk when autism and ADHD traits co-occur, while positive childhood experiences have been linked to protective effects, particularly at higher ADHD-trait levels [103].

Neuroimaging evidence implies frontolimbic circuits in adolescent suicidality. Abnormalities in medial prefrontal and anterior cingulate regions are repeatedly observed in suicidal youth. These brain structures are critical for affect regulation and self-referential processing [58]. In a multimodal study of 68 adolescents and young adults with bipolar disorder (26 suicide attempters, 42 non-attempters), attempters showed significantly reduced orbitofrontal and hippocampal gray matter volumes, lower uncinate fasciculus integrity, and diminished amygdala–ventral prefrontal connectivity compared to non-attempters. Notably, only the connectivity measures correlated with suicidal ideation and attempt lethality [104]. In a sample of 94 unmedicated female adolescents (31 attempters, 27 non-attempters with major depressive disorder, and 36 controls), suicide attempters exhibited altered activity in insular and precentral cortices and abnormal coupling with the cingulate cortex [105]. While Liu et al. [105] did not explicitly frame their findings in network terms, converging evidence implicates cingulate, prefrontal, and striatal systems in altered salience attribution and executive control in suicidality [58,106,107].

Early developmental influences may further bias neural systems toward vulnerability. Prospective studies link prenatal maternal distress to long-term alterations in amygdala volume, limbic–prefrontal connectivity, and HPA axis functioning, suggesting that stress biology can shape emotion regulation circuits before adolescence [108]. Longitudinal evidence further indicates that maternal psychological distress during pregnancy is associated with alterations in brain structure, such as smaller overall brain volume and altered cortical thinning, in offspring (including school-aged children) [109]. These early alterations in frontolimbic circuitry and the developing HPA axis may increase susceptibility to affective dysregulation and heightened stress reactivity, both established risk factors for later psychopathology and suicidality. This offers a plausible developmental pathway linking prenatal stress exposure to suicide risk [9,108,109].

Adverse perinatal conditions such as preterm birth, low fetal growth, and pregnancy complications have been associated with increased suicide risk in offspring, although part of this vulnerability appears to reflect shared familial or prenatal environmental factors rather than direct causal effects [110,111].

Collectively, neurodevelopmental diagnoses, trait configurations, and early neurobiological shaping converge on circuitry that is undergoing rapid remodeling during adolescence. This convergence provides insight into why some youths are especially susceptible to suicidal thoughts and behaviors and highlights preventive levers, including positive childhood experiences, that may recalibrate developmental trajectories [58,101,103].

### 4.5. Sleep and Circadian Rhythm Dysregulation

Disturbances in sleep and circadian regulation are consistently associated with suicidal ideations and behaviors in young people, independent of co-occurring depression [112]. In a large longitudinal cohort of preadolescents, parent-reported sleep problems like nightmares and excessive daytime sleepiness in particular predicted suicidal ideation and attempts over a two-year follow-up, even after adjusting for baseline affective symptoms [113]. Proposed mechanistic models suggest that circadian disruption and insufficient sleep may influence serotonergic signaling, prefrontal–limbic regulation, and impulsivity. However, these pathways remain hypothetical and have not been causally established [114]. Because sleep problems are common and modifiable, their assessment and treatment represent accessible clinical targets to potentially reduce near-term suicide risk [112,113,114].

## 5. Biologically Informed Treatment and Prevention Methods

Guided by the thesis that biologically informed, mechanism-targeted strategies can reduce suicidal risk beyond nonspecific symptom relief, recent evidence highlights three complementary avenues: rapid glutamatergic modulation, longer-term neuroprotective agents, and inflammation- or redox-guided interventions [88,96,115].

Accumulating randomized trials and meta-analyses indicate that ketamine can acutely lower suicidal ideation in adult patients within hours, though durability and optimal maintenance strategies remain inconsistent [115]. Evidence in adolescents is more limited and mixed. In a randomized study, low-dose intravenous ketamine did not outperform placebo on suicidal ideation and produced more dissociation [116]. By contrast, small uncontrolled case series of ketamine-assisted psychotherapy have described rapid improvements, including reductions in suicidal thoughts and self-harm urges in some patients [117]. Ongoing trials are examining whether repeated ketamine infusions combined with psychotherapy can sustain benefits during the high-risk post-discharge period for patients aged 14 to 30 [118]. Esketamine nasal spray is clinically used in adults for treatment-resistant depression. However, its implementation in the younger population is limited, as evidence is restricted to small exploratory studies [119], and it lacks pediatric regulatory approval [91].

The therapeutic mechanisms of established long-term anti-suicidal agents, namely lithium and clozapine, are increasingly understood to involve the modulation of neurobiological pathways mentioned in this paper [115,120]. In pediatric populations, evidence for their anti-suicidal efficacy remains limited to correlational and ecological data, with no randomized controlled trials available [120,121,122,123]. Ecological and registry analyses have reported inverse associations between regional prescribing of lithium and clozapine and adolescent suicide mortality, particularly among males. However, the strength and robustness of these associations vary across studies. A French ecological analysis found that the association remained significant for clozapine after multivariable adjustment, whereas findings for lithium did not persist in adjusted models [120,121,122].

Based on links between suicidal risk, inflammation-driven kynurenine pathway, and glutamatergic dysregulation, some possible prevention and treatment strategies have been proposed, for example, stratification by inflammatory/kynurenine signatures (e.g., IL-6, QUIN/KYNA) and repurposing of anti-inflammatory or microglia-modulating agents (minocycline, cytokine blockade, IDO-1 inhibition). At present, these approaches remain exploratory, and their efficacy and safety in pediatric populations have not been established [87,96].

Pediatric intervention studies add practical weight. Omega-3 polyunsaturated fatty acid supplementation has been shown to alter kynurenine/serotonin balance and improve antioxidant capacity in depressed adolescents. Although baseline levels of BDNF and GPx were correlated with biomarkers of depression in these youths, the supplementation itself did not produce a statistically significant change in their activity [88,95,99]. These findings align with broader Slovak contributions that highlight redox imbalance and inflammation as key biological vulnerabilities in youth [92]. Moreover, oxidative-stress biomarkers such as altered SOD, NO, and uric acid are increasingly identified as correlates of suicide attempt risk in adolescent MDD, opening avenues for biomarker-guided risk stratification [76].

## 6. Discussion

The neurobiology of suicidal behavior in youth reflects a complex interplay of stress reactivity, inflammation, and downstream molecular alterations that differ in several aspects from adult pathophysiology. While research has highlighted key biological pathways, the evidence base is constrained by methodological limitations and a persistent gap between experimental findings and clinical application.

Current data suggest a cascade of biological changes that heighten suicide risk in vulnerable young people [9]. Within the broader suicide literature, dysregulation of the HPA axis and serotonergic signaling have received the most extensive empirical support as components of biological vulnerability, although much of this evidence derives from adult samples [53]. Although HPA abnormalities are consistently reported, cortisol results vary. Some studies indicate hyperactivity, while others (particularly in adolescents) show attenuated responses after chronic stress exposure [9,76]. Other biological vulnerabilities with emerging evidence, including redox and inflammatory imbalance, may amplify cognitive states of defeat and entrapment, thereby linking depressive symptomatology to suicidal ideation in adolescents [53,124]. Elevated inflammatory markers, such as a higher monocyte-to-lymphocyte ratio in MDD patients with recent suicide attempts, further seem to support immune involvement [125]. Psychosocial stressors can trigger both HPA axis disruption and low-grade neuroinflammation [9,99].

Inflammatory activation is believed to induce IDO activity, diverting tryptophan metabolism from serotonin toward the kynurenine pathway [99]. This shift reduces serotonergic precursors and increases neuroactive metabolites such as quinolinic acid, an NMDA agonist implicated in excitotoxicity and oxidative stress [43]. Clinical data show altered antioxidant systems, including elevated superoxide dismutase activity [99,125], while Mendelian randomization analyses suggest that genetically lower uric acid (a potent antioxidant) is associated with an increased risk of suicide attempts [76]. These molecular alterations are believed to contribute to dysfunction in circuits regulating emotion and cognition. Reduced BDNF is frequently reported in suicidality, and in pediatric samples, BDNF correlates with oxidative stress indices, consistent with impaired plasticity [8,43,99]. Importantly, evidence indicates that adolescent metabolic and inflammatory profiles diverge from adult patterns, reinforcing the need for youth-specific models [91,112].

Therapeutic evidence illustrates these differences. In adults, lithium, ketamine, and clozapine demonstrate substantial anti-suicidal efficacy in mood disorders and schizophrenia [43,115]. In younger populations, lithium and ketamine show preliminary signals of benefit, but findings are limited to small, heterogeneous studies and remain insufficient without further randomized trials [119,120]. The dynamic neurobiology of adolescence likely influences stress responsivity and treatment effects [112]. This developmental context may partly explain epidemiological patterns observed in Slovakia and globally, where adolescent females attempt suicide at high rates, but mortality remains lower than in older men [10,12]. Neurodevelopmental disorders such as ASD and ADHD add further risk, linked to impulsivity, emotion dysregulation, and heightened vulnerability to adverse childhood experiences [101,102,103,126].

Methodological shortcomings hamper research progress. Most studies remain cross-sectional, limiting causal inference [19,20]. While some longitudinal work is emerging, such as ADHD-focused cohorts [102], these remain rare. Biomarker research is constrained by small samples, inconsistent diagnostic criteria, a lack of standardized assays, and the absence of standardized pediatric biomarker thresholds, which collectively make replication and synthesis difficult [99,125]. Pediatric clinical trials face similar obstacles. Underpowered designs and methodological heterogeneity yield inconsistent results for lithium and ketamine [119,120]. Collectively, these issues hinder the development of a reliable and generalizable evidence base.

Despite advances in mapping biological pathways, translation into clinical tools for predicting or preventing suicide in youth remains distant. Biomarkers such as cytokines or oxidative stress markers lack sufficient reliability for individual-level prediction [76]. Bridging this gap requires large, multicenter longitudinal studies with standardized protocols and diverse samples.

At the same time, risk-focused models are inadequate. Evidence indicates that intrapersonal resources like positive self-perceptions and self-worth are prospectively associated with lower subsequent suicidal ideation and attempts [127], while school belonging, peer support, and relationships with non-parent adults provide additional protection [19,103]. Emerging literature suggests possible biological mechanisms through which protective factors may operate. Social and familial support may buffer stress reactivity through modulation of HPA axis activity, potentially involving oxytocin signaling and inflammatory processes [3,109]. Physical activity and adequate sleep have been associated with enhanced neuroplasticity and executive control, possibly via BDNF upregulation and serotonergic stabilization, although suicide-specific evidence remains limited [71,72,112]. Positive childhood experiences may support adaptive prefrontal regulation [103], while omega-3 fatty acids have been proposed to reduce oxidative and inflammatory burden, findings largely derived from depression-focused research rather than suicide-specific trials [72,90,95].

Systematic reviews consistently show that resilience factors are understudied relative to risks, leaving a critical gap [20]. Without integrating protective processes alongside biological vulnerabilities, neurobiological research will have limited clinical utility for suicide prevention in youth.

## 7. Conclusions and Future Directions

The accumulated evidence makes it clear that biology contributes meaningfully to suicide risk in young people. Still, it does so in interaction with developmental, psychological, and social contexts rather than in isolation. Current findings are valuable for understanding vulnerability but are not yet precise enough to guide individual prediction or treatment. The next steps for the field are therefore less about cataloging additional correlates and more about building integrative, longitudinal models that clarify causal pathways and identify when biological signals matter most. This may include repeated-measures designs that track stress reactivity over time, multimodal biomarker panels combining inflammatory, neuroendocrine, and neurobiological indicators, as well as developmentally stratified analyses that account for age-specific variability. Progress will depend on large, collaborative studies that apply standardized methods, bridge biomarkers with lived experience, and move beyond risk to test protective factors such as positive childhood experiences, school belonging, and sleep interventions. Clinical trials in youth must prioritize age-specific designs, combine mechanism-based therapies with psychosocial approaches, and evaluate not only symptom relief but also genuine reductions in suicidal behavior. Ultimately, reducing the risk of death by suicide in children and adolescents will require a synthesis of biological insight, developmental sensitivity, and socio-ecological interventions.

## Data Availability

No new data were created or analyzed in this study. Data sharing is not applicable to this article.

## Abbreviations

The following abbreviations are used in this manuscript:

5-HIAA	5-hydroxyindoleacetic acid
5-HT	Serotonin
5-HTTLPR	Serotonin-transporter-linked promoter region
ACE	Adverse childhood experiences
ADHD	Attention deficit hyperactivity disorder
AOPP	Advanced oxidation protein products
BDNF	Brain-derived neurotrophic factor
CRH	Corticotropin-releasing hormone
CRP	C-reactive protein
CSF	Cerebrospinal fluid
DOPAC	3,4-dihydroxyphenylacetic acid
FAs	Fatty acids
FKBP5	FK506-binding protein 5
GABA	Gamma-aminobutyric acid
GPx	Glutathione peroxidase
GSK-3β	Glycogen synthase kinase 3 beta
GWAS	Genome-wide association studies
HPA	Hypothalamic–pituitary–adrenal
HVA	Homovanillic acid
IDO	Indoleamine 2,3-dioxygenase
IL-6	Interleukin-6
LGBTQ	Lesbian, gay, bisexual, transgender, and questioning
MDD	Major depressive disorder
NCZI	National Health Information Centre
NF-κB	Nuclear factor-κB
NMDA	N-methyl-D-aspartate
NO	Nitric oxide
PIC	Picolinic acid
QUIN	Quinolinic acid
RNS	Reactive nitrogen species
ROS	Reactive oxygen species
SLC6A4	Solute carrier family 6 member 4
SOD	Superoxide dismutase
SSRIs	Selective serotonin reuptake inhibitors
TNF-α	Tumor necrosis factor-α
